# Insights into chloroplast genome structure, intraspecific variation, and phylogeny of *Cyclamen* species (Myrsinoideae)

**DOI:** 10.1038/s41598-022-27163-1

**Published:** 2023-01-03

**Authors:** Lihui Mao, Qingcheng Zou, Zhongshuai Sun, Qing Dong, Xuerui Cao

**Affiliations:** 1 Zhejiang Instiute of Landscape Plants and Flowers, Hangzhou, 311251 Zhejiang China; 2 Zhejiang Provincial Key Laboratory of Plant Evolutionary Ecology and Conservation, Taizhou University, Taizhou, 318000 Zhejiang China

**Keywords:** Evolution, Phylogenetics

## Abstract

Species from the flowering plant genus *Cyclamen* are popular amongst consumers. In particular *Cyclamen persicum* Mill. has been significantly used commercially, and certain small flowering species such as *Cyclamen hederifolium* and *Cyclamen coum* are gradually growing in popularity in the potted flower market. Here, the chloroplast genomes of nine *Cyclamen* samples including four *Cyclamen* species and five varieties of *C. hederifolium* were sequenced for genome structure comparison, White green septal striped leaves related gene screening and DNA molecular markers were developed for phylogenetic analysis. In comparing *Cyclamen* species’ chloroplast genomes, gene content and gene order were found to be highly similar with the length of genomes ranging from 151,626 to 153,058 bp. The chloroplast genome of *Cyclamen* has 128 genes, including 84 protein-coding genes, 36 transfer RNA genes, and 8 ribosomal RNA genes. Based on intraspecific variation, seven hotspots, including three genes and four intergenic regions, were identified as variable markers for downstream species delimitation and interspecific relationship analyses. Moreover, a phylogenetic tree constructed with complete chloroplast genomes, revealed that *Cyclamen* are monophyletic with *Lysimachia* as the closest neighbor. Phylogenetic analyses of the 14 *Cyclamen* species with the seven variable regions showed five distinct clades within this genus. The highly supported topologies showed these seven regions may be used as candidate DNA barcode sequences to distinguish *Cyclamen* species. White green septal striped leaves is common in *C. hederifolium*, however the molecular mechanism of this has not yet been described. Here, we find that the intergenic region rps4-trnT-UGU seems related to white green septal striped leaves.

## Introduction

The plant chloroplast genome is highlight conserved in regards to structure, gene number, and gene composition compared with mitochondrial or nuclear genomes^1^. Due to uniparental inheritance and small genome size, complete chloroplast genomes have been used extensively in phylogenetic analyses and species identification^2,3^.

Distributed in and around the Mediterranean region, the genus *Cyclamen*, within the order Ericales, family Primulaceae, and subfamily Myrsinoideae consists of 24 species^4^. Some species have large flowers such as *C. persicum* Mill. which has been bred for more than 150 years, leading to the wide ranges of color seen today along with significant commercial use. Some species of *Cyclamen* are frost resilient, and some can bloom from late summer until late spring. *C. hederifolium* is native to Italy, southern France, Greece, and Western Turkey. It has large intraspecific variation and strong cold and heat tolerance. *C. hederifolium* as the parent to cross with *C. persicum* is one of the main examples of distant cross breeding of *Cyclamen*^4^.

*Cyclamen* *hederifolium* has small flowers and great variation in leaf color such as white and green stripes, light green, and green with white in center. White and green striped leaf color is special and has high ornamental value, however, the formation mechanism of white and green striped variation remains unknown. The mechanisms for the formation of plant leaf spots can be roughly divided into two categories: (1) environmental factors, such as pathogens and nutritional defects, and (2) genetic mutation^5^. Plastid mutation is considered to be one of the main mechanisms for the formation of striped leaves. Under natural conditions, there is a 0.006–0.3% probability of spontaneous mutation of plastid gene which might result in variegated or striped leaf color^6^.

Some plastid mutants cause chlorotic effects, that is, developmental damage or environmental sensitivity, and showing reversible mottled or albino phenotypes. To date, many leaf color mutants produced by plastid gene mutations have been identified, involving eight plastid genes, including *psbE*, *rbcL*, *infa*, *ycf3*, *matK*, and others. Similarly, a mutation in the *rps4* chloroplast gene coding region was described in the yellowing mutant of Chinese cabbage (Brassica campestris ssp. pekinensis) and named *cdm*, consisting of a Val to Gly conversion in the *rps4* protein’s 193th amino acid. qRT-PCR showed that the expression level of the *rps4* gene was down-regulated in *cdm*^7^. The candidate chloroplast genes related to the formation of white green septal striped leaves may be identified through comparative analyses of chloroplast genomes.

In contrast, the taxonomy of *Cyclamen* remains unclear. Phylogenetic analyses of Primulaceae, Myrsinaceae, and Theophyllastaceae based on three chloroplast gene fragments revealed that the clade composed of *Aegiceras*, *Grammadenia*, *Myrsine*, and *Hymenandra* has the closest genetic relationship with *Cyclamen*, but the nodes of phylogenetic tree showed a comb structure and lacked support rates^8^. In contrast to the phylogenetic reconstructions based on plastid gene sequences, when using ITS sequences, *Cyclamen* does not appear as a member of the Myrsinaceae-Lysimachieae clade. Therefore, *Cyclamen*’s exact position on the phylogenetic tree remains unclear^9^.

A phylogenetic tree constructed with only one gene or several genes has low reliability, and the phylogenetic tree of Myrsinaceae based on chloroplast genomes did not include the genus *Cyclamen*^10^. Therefore, little is known about the evolutionary relationships between species within the *Cyclamen* genus. *Cyclamen* a resource rich genus, and its value and application covers many aspects, such as ornamental, fragrance, and medicine, and thus the study of intraspecific relationships is of great significance for cross breeding. In recent years, the improvement of sequencing technology and decrease in costs has led to omics data from many systems, however, there genetic data from this genus remains scarce, which thus hinders basic research at a molecular level.

In this work, we sequenced and assembled nine *Cyclamen* whole chloroplast (cp) genomes and performed a comprehensive analysis, including genome features comparisons, repeats, selective pressures, divergence hotspots, and phylogenetic relationships. Our goals in this study were to: (1) Present the complete cp genome sequences of nine newly assembled nine *Cyclamen* samples in five species and construct phylogenetic tree of Myrsinaceae; (2) Identify candidate chloroplast genes related to the formation of white green septal striped leaves through a comparative analysis of the chloroplast genome; (3) identify divergence hotspots as potential genetic markers for DNA barcoding and test the effectiveness of these markers; and (4) construct the phylogeny of 14 *Cyclamen* species using selected DNA markers and explore their interspecific relationships.

## Results

### Chloroplast genome sequencing and features of cyclamen species

**Figure 1:**
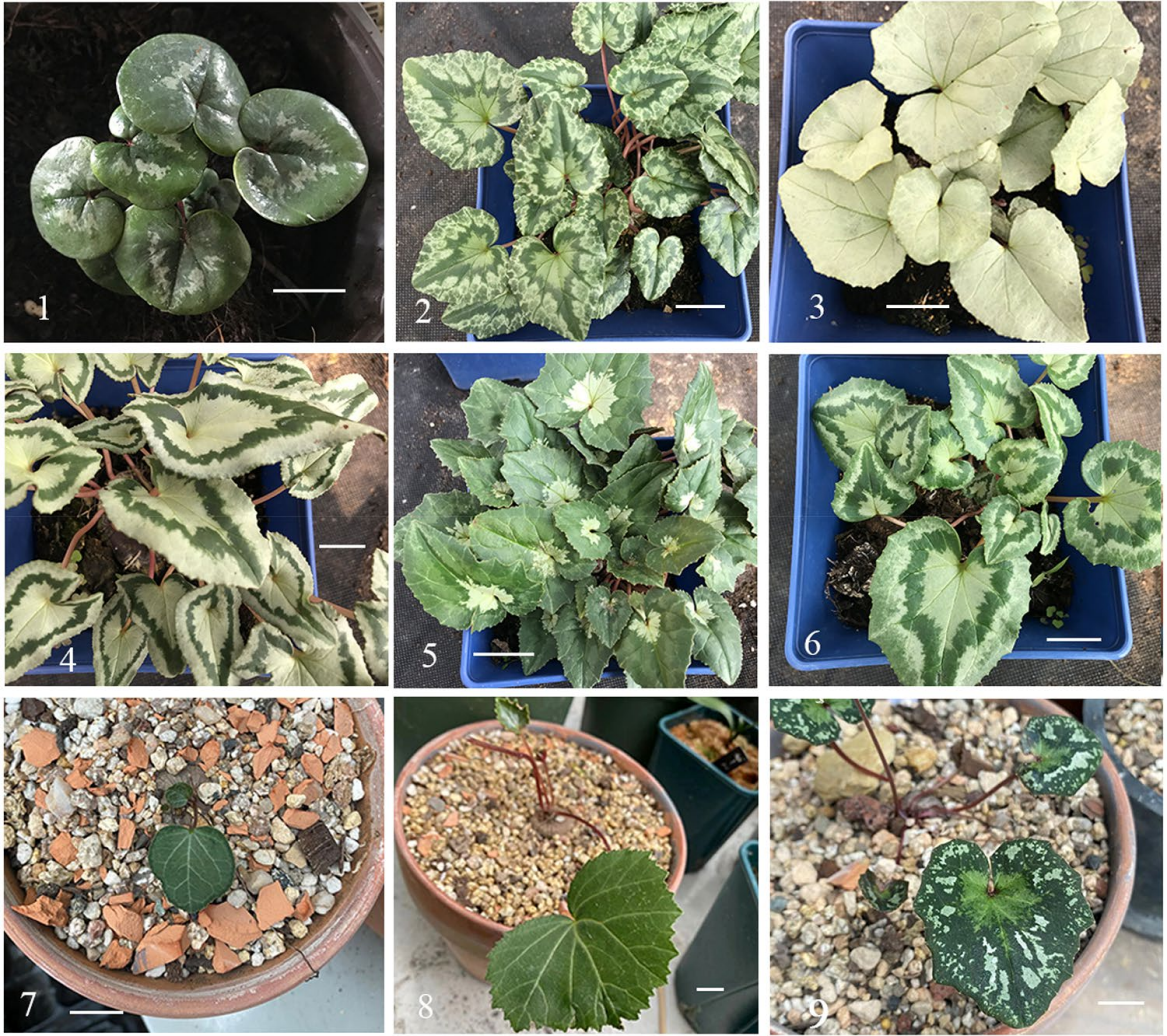
Nine sequenced samples in this study. (1) *C. coum*, (2) *C. hederifolium*-1, (3) *C. hederifolium*-2, (4) *C. hederifolium*-3, (5) *C. hederifoliu*m-4, (6) *C. hederifolium*-5, (7) *C. graecum*, (8) *C. rohlfsianum*, (9) *C. cyprium* (all bars are 2 cm).

**Figure 2:**
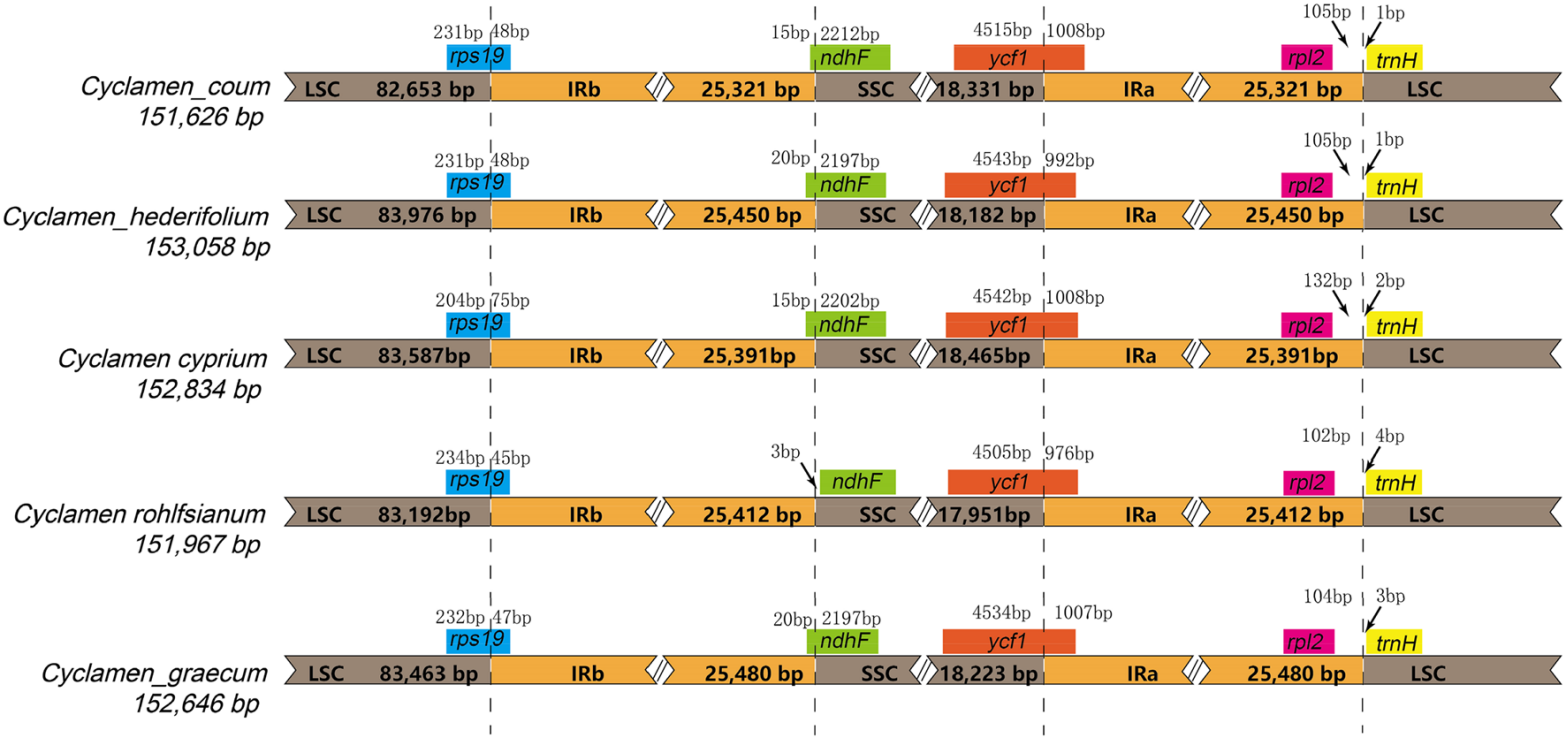
Comparison of the borders of LSC, SSC, and IR regions among five *Cyclamen* species.

Nine complete chloroplast genome sequences represent five species were deposited in GenBank with the accession numbers: ON480518, ON480519, ON480520, ON480521, ON480522, ON480523, OP957067, OP957068 and OP957069. The assembly results were uniform and the validating of the assembly results by mapping reads to the assembled sequence were showed in supplementary materials (Supplementary Fig. S1). The total chloroplast genome size ranged from 151,626 bp (*C. coum*) to 153,058 bp (*C. hederifolium* with white green septal striped leaves) (Fig. 1). The Cyclamen chloroplast genome has a typical quadripartite structure and includes a pair of IR regions (25,321–25,480 bp), LSC regions (82,653–83,976 bp), and SSC regions (18,182–18,465 bp) (Fig. 2).

### Long repeat and SSR analysis

**Figure 3:**
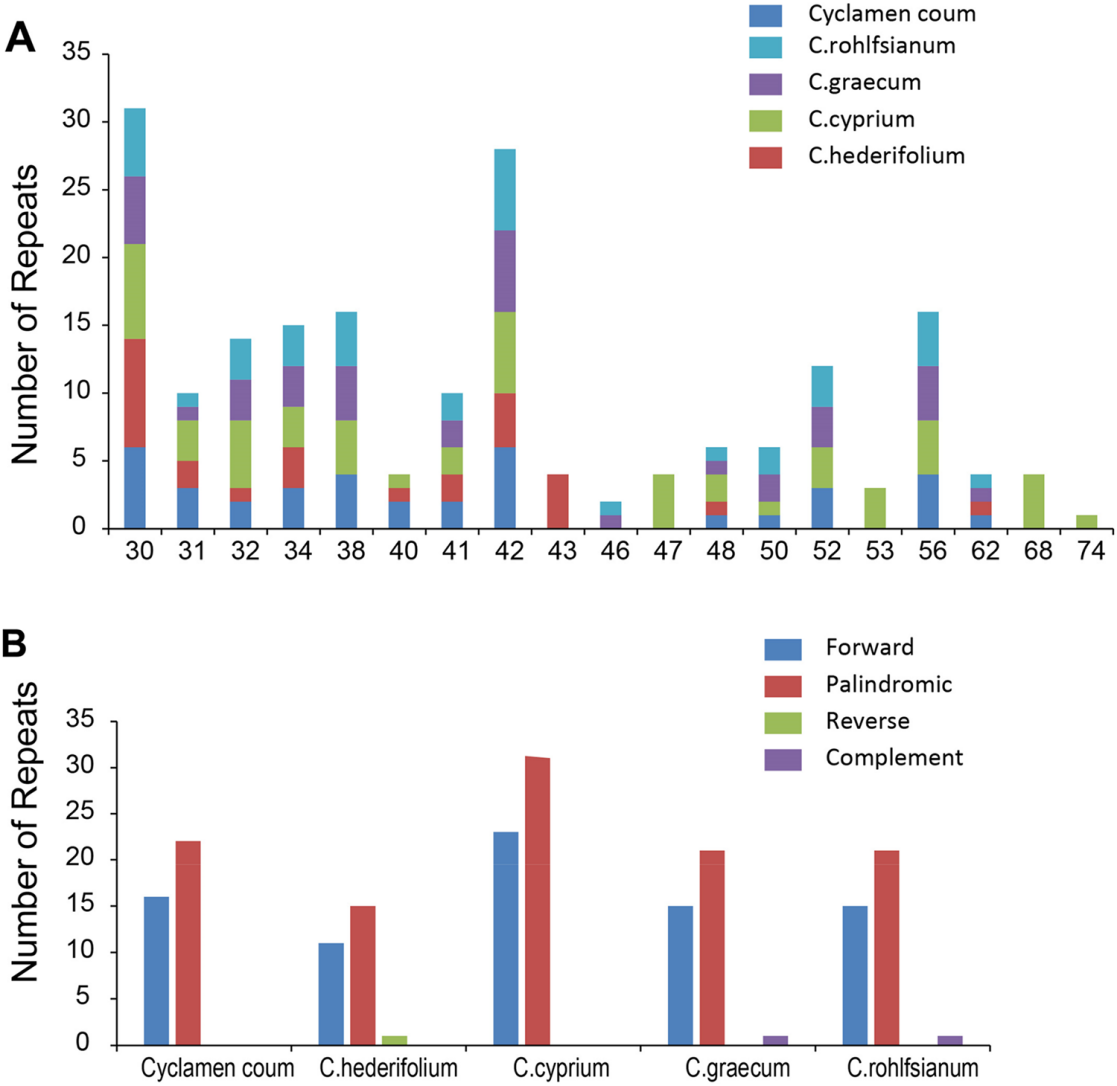
The number of long repeats in the whole cp genome sequence of *C. coum* and *C. hederifolium. *(**A**) Frequency of repeats more than 30 bp long. (**B**) Frequency of repeat type.

Repeat sequences in *Cyclamen* cp genomes were detected with REPuter (https://bibiserv.cebitec.uni-bielefeld.de/reputer), with a copy size of 30 bp or longer. 38 long repeats consisting of 16 forward repeats and 22 palindromic repeats were detected in *C. coum* while 27 long repeats consisting of 11 forward repeats, 15 palindromic repeats, and 1 reverse repeat were detected in *C. hederifolium* (Fig. 3). Five *C. hederifolium* samples shared the same SSR features, but there is some variation between the five *Cyclamen* species in SSR number and type (Table 1).

**Table 1:** SSR type of the five *Cyclamen* species.

SSR type	*C. coum*	*C. hederifolium*	*C. cyprium*	*C. graecum*	*C. rohlfsianum*
A/T	54	51	54	46	36
AT/AT	7	8	8	6	5
AAC/GTT	7	7	6	7	7
AAG/CTT	24	25	24	24	24
AAT/ATT	18	22	20	24	26
ACT/AGT	1		1	1	1
AGC/CTG	7	7	7	7	6
AGG/CCT	2	2	2	2	2
ATC/ATG	4	5	4	5	5
AAAG/CTTT	1	1	1	1	1
AAAT/ATTT	4	3	4	4	2
AAGG/CCTT		1	1	1	1
AATC/ATTG	1	1	1	1	1
AATT/AATT	1	1	1	1	1
AAATT/AATTT	1				
AATAG/ATTCT				2	1

The chloroplast genome of *Cyclamen* has 128 genes, including 84 protein-coding genes, 36 transfer RNA genes, and eight ribosomal RNA genes. Six protein-coding genes (*rps7*, *rps12*, *rpl2*, *rpl23*, *ndhB*, and *ycf2*), seven tRNA genes (*trnA-UGC*, *trnI-GAU*, *trnI-CAU*, *trnL-CAA*, *trnN-GUU*, *trnR-ACG*, and *trnV-GAC*) and all four rRNA genes are duplicated in the IR regions. Fourteen genes (*trnA-UGC*, *trnI-GAU*, *trnK-UUU*, *trnL-UAA*, *trnV-UAC*, *rps12*, *rps16*, *rpl2*, *rpl16*, *rpoC1*, *petB*, *petD*, *atpF*, *ndhA*, and *ndhB*) contain a single intron and two genes (*clpP* and *ycf3*) have two introns (Table 2).

**Table 2:** List of annotated genes in *Cyclamen* chloroplast genomes.

Gene group	Gene name
Ribosomal RNAs	*rrn16(2), rrn23(2), rrn4.5(2), rrn5(2)*
Transfer RNAs	trnA-UGC*(2), trnC-GCA, trnD-GUC, trnE-UUC, trnF-GAA, trnfM-CAU, trnG-GCC, trnH-GUG, trnI-GAU*(2), trnI-CAU(2), trnK-UUU*, trnL-CAA(2), trnL-UAA*, trnL-UAG, trnM-CAU, trnN-GUU(2), trnP-UGG, trnQ-UUG, trnR-ACG(2), trnR-UCU, trnS-GCU, trnS-GGA, trnS-UGA, trnT-GGU, trnT-UGU, trnV-GAC(2), trnV-UAC*, trnW-CCA, trnY-GUA
Small ribosomal subunit	rps2, rps3, rps4, rps7(2), rps8, rps11, rps12*(2), rps14, rps15, rps16*, rps18, rps19
Large ribosomal subunit	rpl2*(2), rpl14, rpl16*, rpl20, rpl22, rpl23(2), rpl32, rpl33, rpl36
DNA dependent RNA polymerase	rpoA, rpoB, rpoC1*, rpoC2
Photosystem I	*psaA, psaB, psaC, psaI, psaJ*
Photosystem II	*psbA, psbB, psbC, psbD, psbE, psbF, psbH, psbI,* *psbJ, psbK, psbL, psbM, psbN, psbT, psbZ*
Cytochrome b/f complex	petA, petB*, petD*, petG, petL, petN
Subunits of ATP synthase	atpA, atpB, atpE, atpF*, atpH, atpI
Protease	clpP**
Large subunit of rubisco	*rbcL*
NADH dehydrogenase	ndhA*, ndhB*(2), ndhC, ndhD, ndhE, ndhF, *ndhG, ndhH, ndhI, ndhJ, ndhK*
Maturase	*matK*
Envelope membrane protein	*cemA*
Acetyl-CoA carboxylase	*accD*
c-type cytochrome Synthesis gene	*ccsA*
Open reading frames(ORF, *ycf*)	ycf1, ycf2(2), ycf3**, ycf4

*Indicates gene with one intron and **indicates gene with two introns. (2) indicates that the number of the repeat unit is 2. The *rps12* gene is a trans-spliced gene.

### Phylogenetic analysis reveals inter- and intraspecific variation

**Figure 4:**
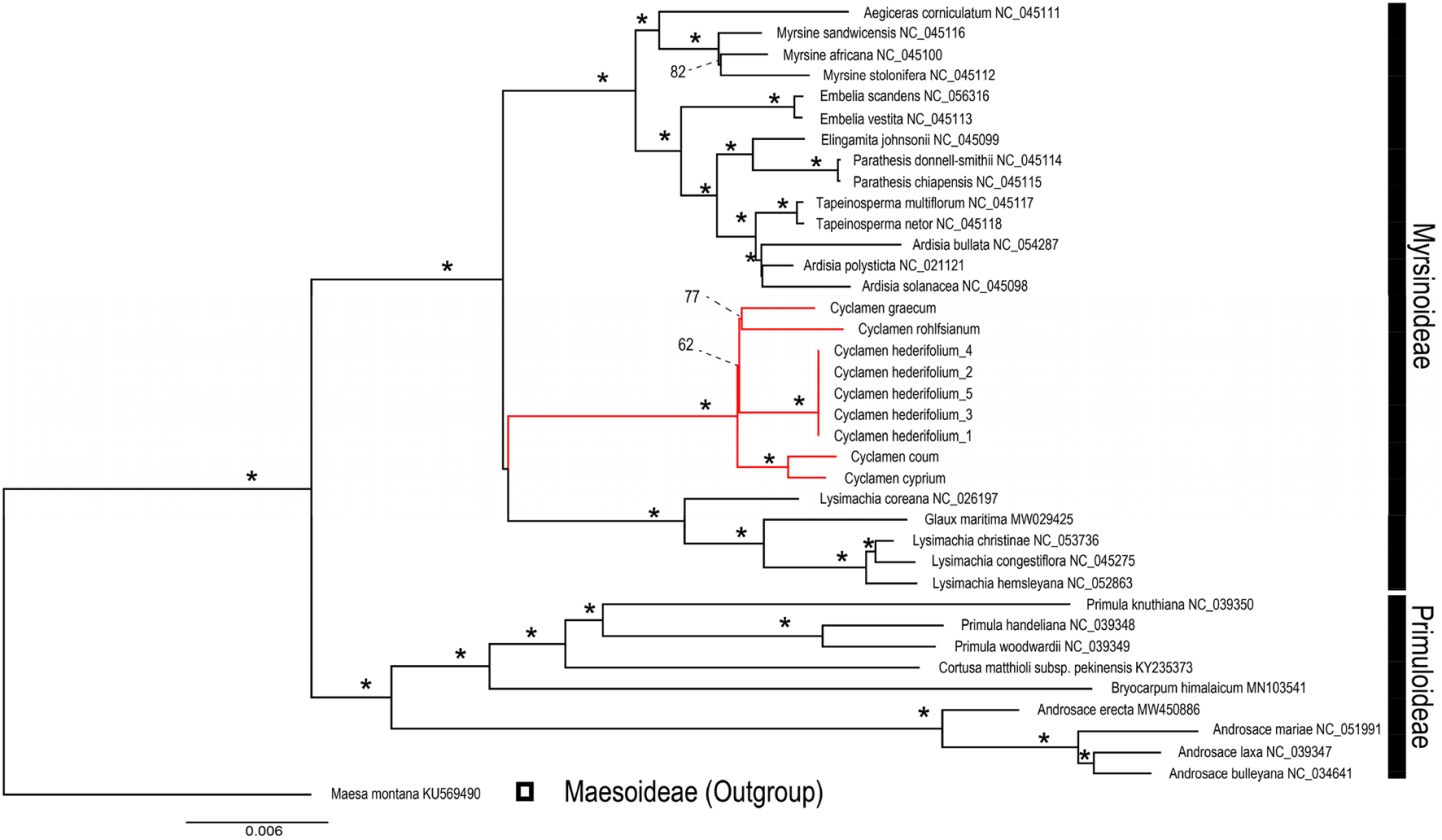
Phylogenetic tree constructed using maximum likelihood method based on the whole chloroplast genomes of 33 different species (The symbol * in the phylogenetic tree indicated that the support value is 100%).

A total of 28 Myrsinoideae and 9 related cp genomes were included in the phylogenetic analysis. The molecular phylogenetic tree showed the *Cyclamen* is monophyletic, located in Myrsinoideae and is closely related to the genera *Lysimachia* and *Glaux* (Fig. 4). Ten genera in Myrsinoideae formed four clades, one on them includes *Aegiceras* and *Myrsine*. The second consists of *Embellia*, *Ardisia*, *Tapeinosperma*, and *Elingamita*. *Cyclamen* represents one clade and the fourth clade includes *Lysimachia* and *Glaux*. In particular, *Lysimachia* and *Glaux* are not reciprocal monophyly: *Glaux* is embedded in *Lysimachia* which indicates that accurate classification of these two genera requires further study.

**Figure 5:**
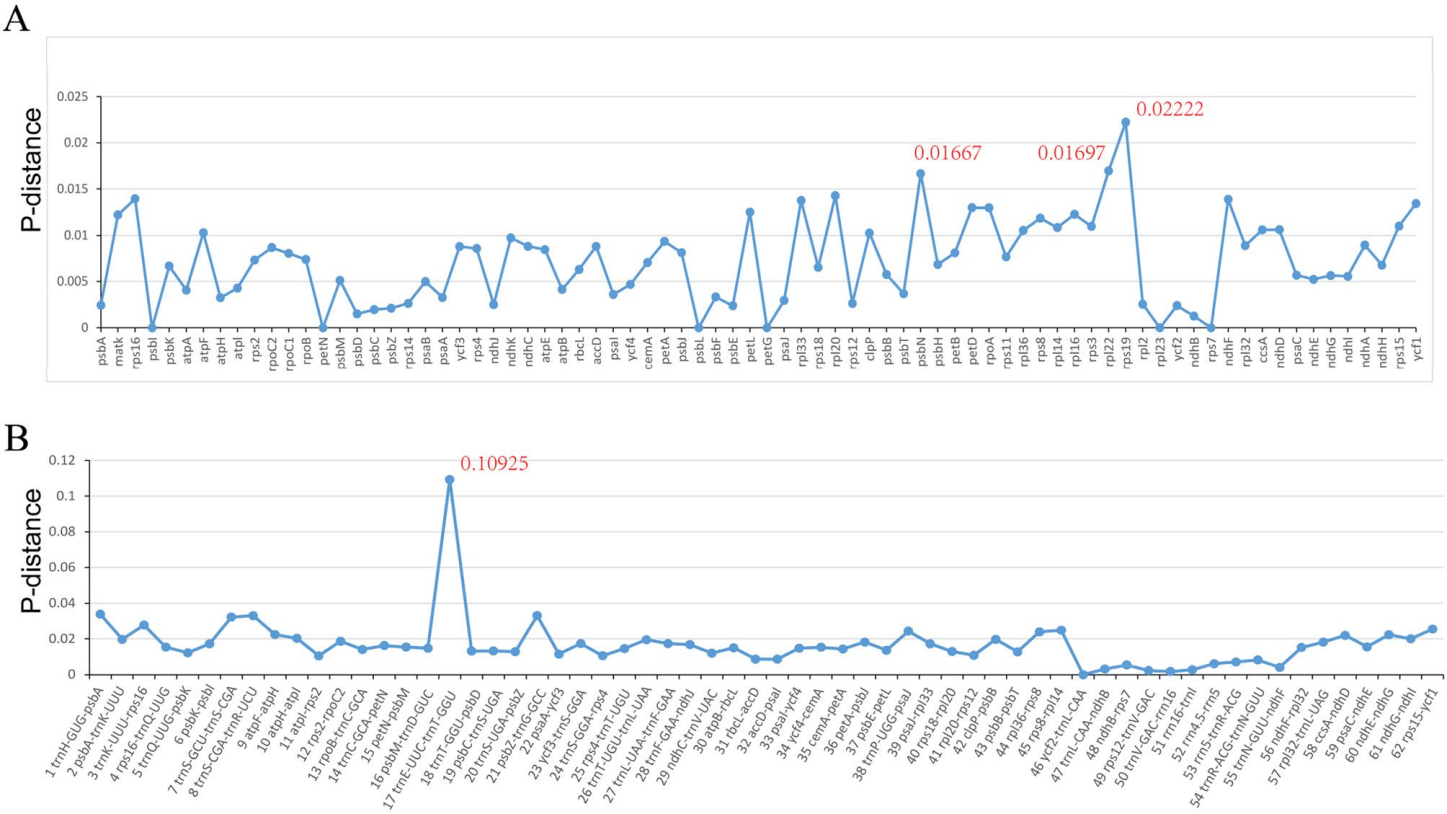
Nucleotide variability (Pi) values were calculated in the five *Cyclamen* species chloroplast genomes. (**A**) Protein-coding genes. (**B**) Intergenic regions.

We analyzed the nucleotide diversity (Pi) values to measure the divergence levels of protein-coding genes and intergenic regions of the five *Cyclamen* species. The level of sequence divergence among protein-coding genes was more conserved than in intergenic regions. The Pi value was from 0 to 0.02222 in protein-coding genes while it ranged from 0 to 0.10925 in intergenic regions (Fig. 5). Three genes and four intergenic regions were selected for interspecific relationship analysis due to their relatively high Pi value and potential success as PCR primers (Table 3).

**Table 3:** The variability of the hypervariable markers.

Sequence name	Length range	Variable sites	Variable sites (%)	Nucleotide diversity
*rpl22*	448	38	8.48	0.0184
*ndhF*	960	73	7.60	0.0177
*Ycf1-1*	1341	89	6.64	0.0148
*Ycf1-2*	1309	104	7.94	0.0179
*Ycf1-3*	1376	110	7.99	0.0161
*rpoB-trnC*	696	43	6.18	0.0131
*trnS-rps4*	275	16	5.82	0.0113
*rps4-trnT*	278	14	5.04	0.0118
*ycf4-petA*	1042	53	5.09	0.0106

**Figure 6:**
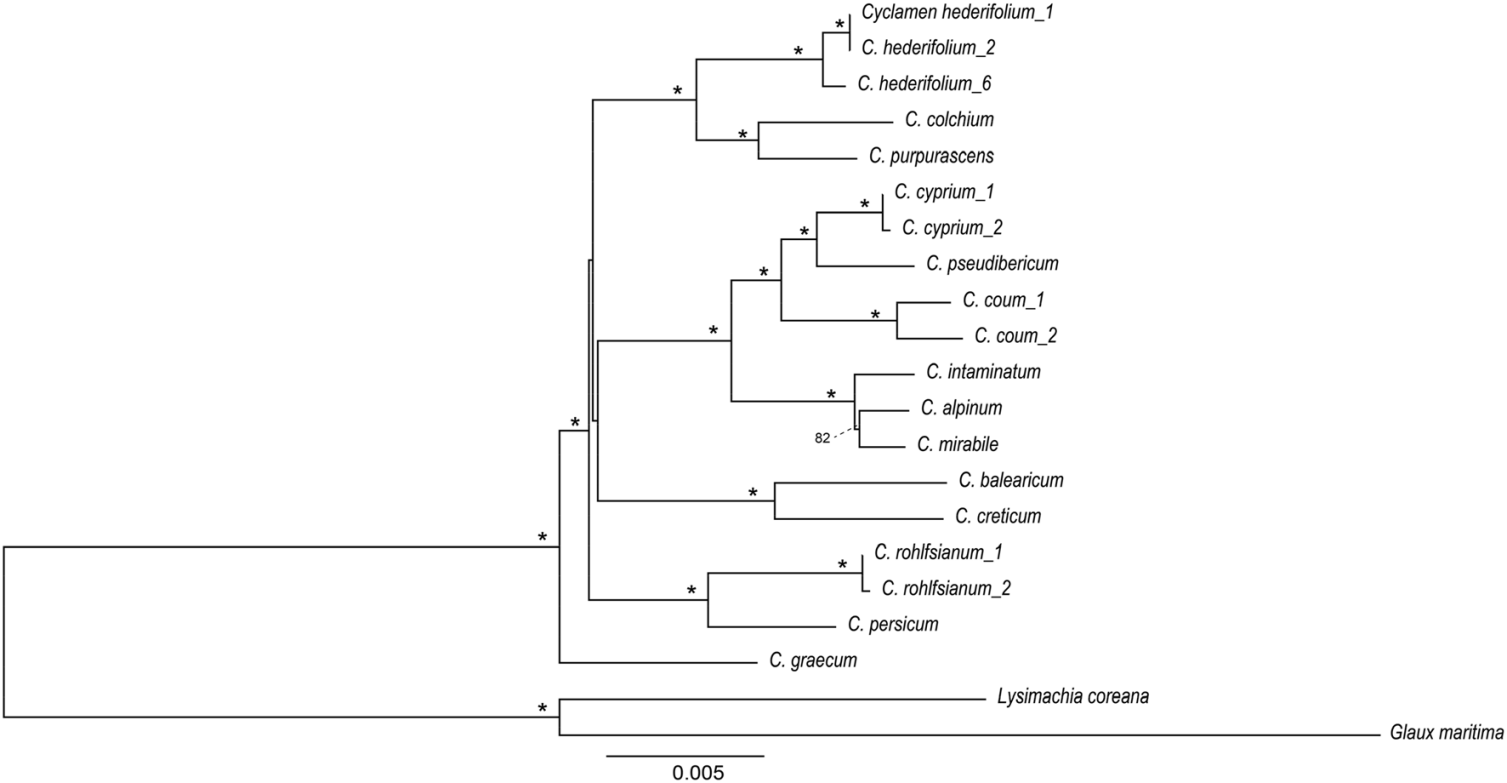
Phylogenetic relationship among the 14 *Cyclamen* species based on nine seven segments (The symbol * in the phylogenetic tree indicated that the support value is 100%).

The phylogenetic analysis of the 19 samples represent 14 species was constructed based on three genes (*Ycf1* was divided into three fragments as it is too long to amplify and sequence) and four intergenic regions. Different samples of same species like *C. hederifolium*, *C. cyprium*, *C. coum* and *C. rohlfsianum* formed separated branches indicating the effectiveness of these genes in phylogenetic construction. 14 species in *Cyclamen* genus formed five clades, one of which includes *C. hederifolium, C. colchium* and *C. purpurascens*. The second clade is comprised of *C. cyprium*, *C. pseudibericum*, *C. coum*, *C. intaminatum, C. alpinum* and *C. mirabile*. The third clade consisted of *C. creticum* and *C. balearicum*. The fourth clade includes *C. rohlfsianum* and *C. persicum*. The species *C. graecum* formed the fifth clade (Fig. 6).

The gene *rpl22* has high interspecific variation and low intraspecific variation in *Cyclamen*. The amplified length of this gene is 448 bp, with 38 basepair variations within the genus. This gene can identify species with a single DNA sequence, which is simple, efficient, and low cost (Supplementary Fig. S2).

## Discussion

In this study, we sequenced and annotated the cp genomes of nine *Cyclamen* samples, compared genomic features among the samples of *Cyclamen*, constructed cp genome phylogenetic tree to analyze the location of the genus, and identified SSR, long repeats and suitable polymorphic loci of suitable molecular markers for phylogenetic analysis. Using the complete chloroplast genome phylogenetic inference of the nine *Cyclamen* samples we annotated and the 24 species from Myrsinoideae and nine Primuloideae downloaded from NCBI, helped to elucidate the taxonomic position of the genus *Cyclamen* within Myrsinoideae and in close relationship with the clade formed by *Lysimachia* and *Glaux*.

The cp genomes of *Cyclamen* species have 128 genes, including 84 protein-coding genes, 36 transfer RNA genes, and eight ribosomal RNA genes. Only one insertion/deletion mutation in the intergenic region *rps4-trnT-UGU* among the five *Cyclamen hederifolium* chloroplast genome samples was described. This insertion/ deletion seems to be associated with white green septal striped leaves in samples *C. hederifolium1*, *C. hederifolium3*, and *C. hederifolium5* which have the -ATCAATAATT insertion have special leaf variegation. In contrast, samples *C. hederifolium2*, *C. hederifolium4*, and other four species lack the -ATCAATAATT insertion and do not have white green septal striped leaves. Variegated-leaf plants are ideal materials for studying the mechanism of chloroplast development and interaction between plastid and nucleus, because of the coexistence of green sectors with normal chloroplast development and white or yellow sectors with abnormal chloroplast development in the same leaf^11^. In this research, insertion/deletion mutations in the intergenic region *rps4-trnT-UGU* were screened to be related to white green septal striped leaves, however, the mechanism of interaction between chloroplast development and nucleus remains unknown.

The entire cp genome may be used directly as a long barcode for species identification and phylogenetic analysis. Furthermore, the hypervariable regions described here may be screened out as potential molecular markers for species identification^12,13^.

The phylogenetic tree based on the whole cp genome showed the *Cyclamen* is monophyletic, in the family Myrsinoideae and closely related to the genera *Lysimachia* and *Glaux*. Ten genera of Myrsinoideae formed four clades, one on them including *Aegiceras* and *Myrsine*. The second consists of *Embellia*, *Ardisia*, *Tapeinosperma,* and *Elingamita*, which compose the Myrsinaceae s.str. clade which is a tropical woody representative in Myrsinoideae of Primulaceae^10^. *Cyclamen* represents one clade and the fourth clade includes *Lysimachia* and *Glaux*. In particular, *Lysimachia* and *Glaux* do not exhibit reciprocal monophyly; *Glaux* is embedded into *Lysimachia* which indicates that the classification of these two genera needs further investigation.

The repeat variations in plastid genomes are often involved in diverse cellular functions including gene evolution, RNA editing, and gene mobility^14^. cpSSR usually showed high polymorphism in close related species and within the same species, and which are potentially useful markers for interspecific relationship and population genetics^15^. Here, SSRs are varying in number and type between five *Cyclamen* species, and the most abundant repeat type was found to be A/T. The enriched SSRs in five genomes to be tri-nucleotides, followed by mononucleotides, tetra-nucleotides, dinucleotides and and penta-nucleotides. cpSSRs derived from five *Cyclamen* species are expected to be useful for the genetic diversity and molecular breeding studies.

Three genes and four intergenic regions including *rpl22*, *ndhF*, *Ycf1*, *rpoB-trnC*, *trnS-rps4*, *rps4-trnT*, and *ycf4-petA* can be successfully amplified and are also effective in phylogenetic analysis. The phylogenetic tree shows that the genetic relationship between individuals within a species is closer than that between species, and each branch has a high support rate, which indicates that the phylogenetic tree has high reliability. *C. creticum* is closely related to *C. balearicum*, a species endemic to the Balearic Islands and southern France^16,17^. Research based on ITS, *trnL-F*, and *rps16* also showed that *C. creticum* and *C. balearicum* are closely related; that *C. hederifolium, C. colchicum*, and *C. purpurascens* have close genetic affinity; that *C. cyprium* and *C. pseudibericum* are positioned within the same clade; and that *C. intaminatum* is closely related to *C. mirable*^18^. Our phylogenetic tree based on seven fragments are consistent with these previous findings, supporting that the tree generated in our study is reliable. It also bears mention that the a majority of branches in the phylogenetic tree of *Cyclamen* of our study have 100% support, again indicating that the tree is reliable.

DNA barcoding is simple, accurate, and fast, and is therefore the most effective method for species identification^19,20^. Sequences with high success rates of amplification and sequencing, high variability among species but low nucleotide variation within species (less than 2%) are considered to be excellent DNA markers^21^. The *rpl22* gene is efficient for species identification in the genus as it can identify species with a single gene, as it has high rates of variable sites (8.48%) within genus and only 0.45% nucleotide variation within species.

Our results enrich the data on the cp genomes of the genus *Cyclamen* and shed light on the phylogenetic relationship between genera and species. Here, candidate chloroplast genes related to the formation of white green septal striped leaves have been identified through comparative analysis of chloroplast genomes. This work lays an important foundation for molecular breeding and cross breeding research.

## Methods

### Chloroplast plant material and DNA extraction

The leaves of Cyclamen species were obtained from a personal gardener in Chiba-ken, in Japan (Fig. 6). Total genomic DNA was extracted from leaf material with the plant genomic DNA kit (Tiangen Biotech, Beijing, China) according to the manufacturer’s protocol. The final DNA concentration (> 50 ng/μL) was measured using a NanoDrop spectrophotometer, and fragmentation was achieved using sonication.

### Sequencing, assembly, and annotation

First, the fragmented DNA was purified and end-repaired, and sizes were determined by gel electrophoresis. Paired-end libraries with insert sizes of 350 bp were prepared following Illumina’s standard genomic DNA library preparation procedure, and subsequently, a control library quality for sequencing was prepared. Whole genome samples were sequenced on a Illumina Novaseq 6000 platform (Illumina, USA).

Paired-end Illumina raw reads where cleaned and quality filtered using Trimmmatic with the following settings: trim from both ends, remove individual bases with Phred quality score < 20, remove read if three consecutive uncalled bases^22^. Entire reads with a median quality score lower than 21 or less than 40 bp in length after trimming were discarded. After quality filtering, reads were mapped using Bowtie2 v.2.2.6 to the chloroplast genome of the closest related species in order to exclude reads of nuclear and mitochondrial origins^23^. Subsequently, all putative chloroplast reads which mapped to the reference sequence were de novo assembled to reconstruct the chloroplast genomes using GetOrganelle 1.7.5^24^. Finally, clean reads were mapped to the complete plastome again to examine and correct mis-assemblies manually. Automatic annotation of chloroplast genomes was generated with CpGAVAS2^25^ and manually corrected by referring to previously published plastomes on Geneious^26^. Finally, a circular representation of both sequences was drawn using the online tool OGDRAW^27^ (https://chlorobox.mpimp-golm.mpg.de/OGDraw.html).

### Comparison of the chloroplast genomes and divergent hotspot identification

The chloroplast genome composition of LSC, SSC, and IRs were compared according to their annotations. Genome rearrangements were performed with the Mauve Genome Alignment v2.3.1^28^ Plugin and the progressive Mauve algorithm^29^.

### Phylogenetic analysis

Thirty-seven complete chloroplast sequences were used in the phylogenetic analysis, including nine *Cyclamen* samples and 28 samples of other species of Myrsinoideae and Primuloideae from GenBank.

All of the cp genome sequences were aligned using MAFFTv7^30^ and adjusted manually as needed. Maximum likelihood (ML) analyses were implemented with RAxML version 8.2.12^31^. RAxML is based on the general time reversible (GTR) model of nucleotide substitution with the gamma model of rate heterogeneity. Non-parametric bootstrapping was implemented with the fast bootstrap algorithm of RAxML used 1000 replicates.

### Identification of variable DNA markers and phylogenetic relationships within the genus

Variable DNA marker genetic sequences were first aligned using MAFFT v7^30^ and then DnaSP version 5.1^32^ was used to evaluate the nucleotide variability (Pi) of gene. Genes and intergenic regions with high nucleotide variability were used to design primers. Primers were amplified in 14 species of the *Cyclamen* genus to test validity and construct phylogenetic relationships.

### Sample collection and experiment statement

All the methods including plant leaves collection and experiment were carried out in accordance with relevant national/international/legislative and institutional guidelines and regulations.

## Supplementary Information

Supplementary Figures.

## Data Availability

The complete chloroplast sequence generated and analyzed during the current study is available in GenBank (https://www.ncbi.nlm.nih.gov, with the accession numbers: ON480518, ON480519, ON480520, ON480521, ON480522, ON480523, OP957067, OP957068 and OP957069).
