# Supplementary material for: Insights into chloroplast genome structure, intraspecific variation, and phylogeny of Cyclamen species (Myrsinoideae)
Source: Sci Rep. 2023 Jan 3;13:87. doi: 10.1038/s41598-022-27163-1 (PMC9810647; doi:10.1038/s41598-022-27163-1)
Supplement: Supplementary file 1 — Supplementary Figures. [file 41598_2022_27163_MOESM1_ESM.docx]

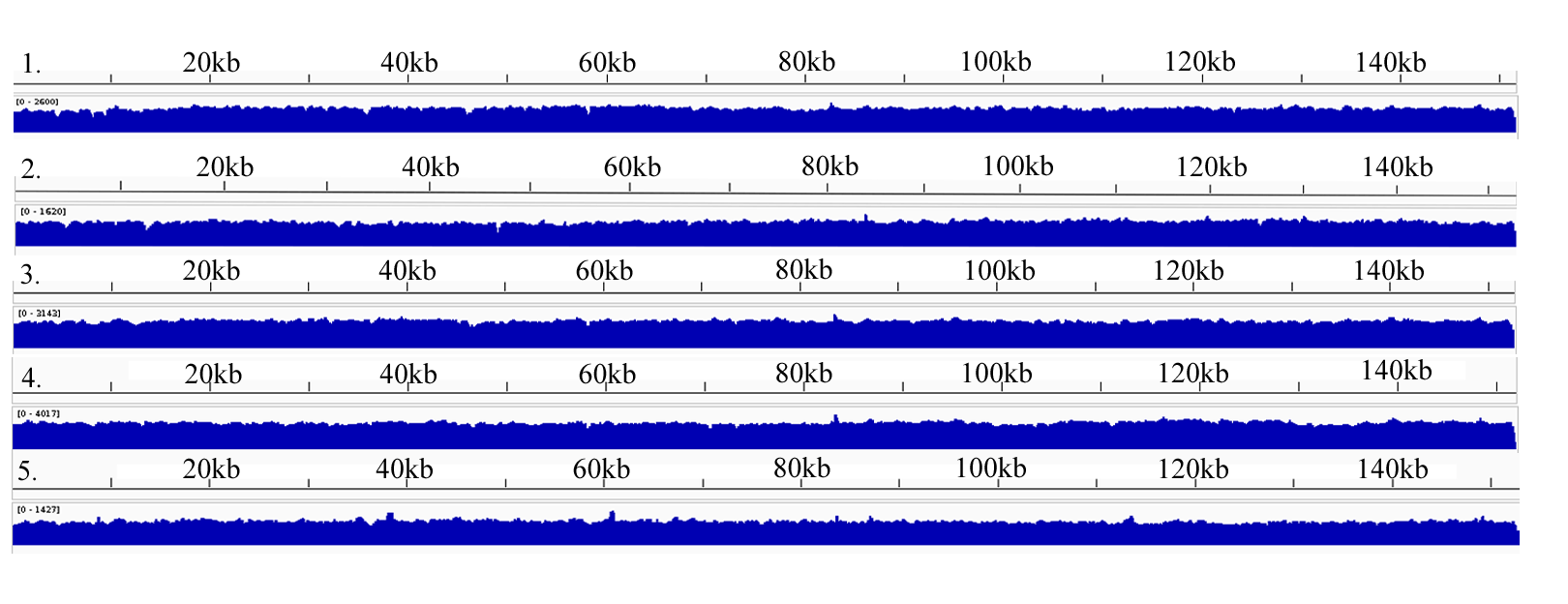


Figure S1 Figures of mapping reads to the assembled *Cyclamen* species.(1. *C. coum*, 2. *C. hederifolium*, 3. *C. graecum*, 4. *C. rohlfsianum*, 5. *C. cyprium*; the height of blue represents mapping depth)


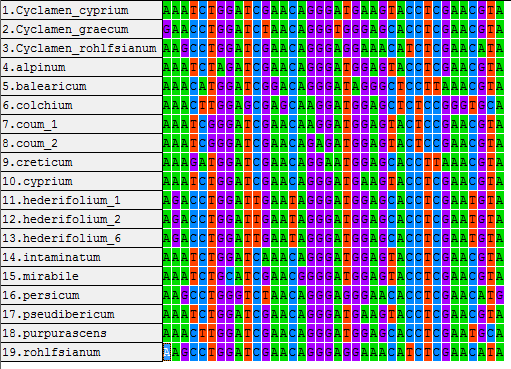


Figure S2 The 38 variation sites of *rpl*22 gene in *Cyclamen*
